# Strong orbital interaction in a weak CH-π hydrogen bonding system

**DOI:** 10.1038/srep22304

**Published:** 2016-03-01

**Authors:** Jianfu Li, Rui-Qin Zhang

**Affiliations:** 1 Department of Physics and Materials Science, City University of Hong Kong, Hong Kong SAR, China

## Abstract

For the first time, the intermolecular orbital interaction between benzene and methane in the benzene-methane complex, a representative of weak interaction system, has been studied by us using *ab initio* calculations based on different methods and basis sets. Our results demonstrate obvious intermolecular orbital interaction between benzene and methane involving orbital overlaps including both occupied and unoccupied orbitals. Similar to interatomic orbital interaction, the intermolecular interaction of orbitals forms “bonding” and “antibonding” orbitals. In the interaction between occupied orbitals, the total energy of the complex increases because of the occupation of the antibonding orbital. The existence of the CH-π hydrogen bond between benzene and methane causes a decrease in rest energy level, leading to at least −1.51 kcal/mol intermolecular interaction energy. Our finding extends the concept of orbital interaction from the intramolecular to the intermolecular regime and gives a reliable explanation of the deep orbital reformation in the benzene-methane complex.

Orbital interaction theory, which provides a comprehensive model for examining the structures and kinetic and thermodynamic stability of molecules, has made a great contribution to our understanding of the fundamental processes in chemistry^1^. Atomic orbitals form molecular orbitals through overlap which leads to a lower energy state of the system. The two new orbitals which are formed from the interaction of two atomic orbitals are the bonding orbital and the antibonding orbital. The energy of the bonding orbital and the energy of the antibonding orbital are respectively lower and higher than that of the original atomic orbitals. As their understanding of orbital interaction theory increased, chemists found that orbital interactions not only exist between atoms but also within special organic molecules through space (TS) and through bond (TB)^2^,^3^,^4^. The concept of TS and TB orbital interactions originally proposed by Gleiter and Hoffmann is very meaningful in demonstrating the interaction in conjugated systems. It has also been applied to analyze reactions^5^, electron transfer^6^,^7^,^8^, and so on. TS orbital interactions occur directly between nearby orbitals, especially between π-π, *p*-π, and σ-π in conjugated systems, and TB interactions result from the coupling effects that occur indirectly through the σ-bond skeleton^2^,^4^,^9^. Both TS and TB orbital interactions occur between close orbitals: the distance between orbital centers is usually less than 3.0 Å^2^,^3^,^4^,^10^.

In the last two decades, the benzene-methane complex has been used as a model system to study the CH-π interaction, which is considered to be a weak hydrogen bond^11^,^12^ and has been found to play important roles in the physical, chemical, and biological properties of a variety of substances^13^,^14^,^15^,^16^,^17^,^18^,^19^,^20^. Hydrogen bonds are formed between two molecules with strongly contrasting electronegativities, one of which is terminated by a hydrogen atom^21^. This type of bonding has been studied for more than a century, and remains to be an active topic in contemporary scientific research. Different from conventional hydrogen bonds, the interaction between benzene and methane has a dual nature in that both dispersion and electrostatic terms contribute to the interaction energy^22^. In the benzene-methane complex, there is strong overlap between the delocalized π conjugation of benzene and the σ bond of methane which is expected to result in an intermolecular TS interaction. However, until now, no research has been conducted on the interaction of intermolecular orbitals in CH-π hydrogen bond systems.

Our previous work found that the CH-π hydrogen bond interaction between benzene and methane has a great impact on the orbital distribution of the HOMO-4 and LUMO+2 of the complex^23^. In previous studies, non-covalent interactions, such as hydrogen-bond interaction, Lone Pair-π interaction and anion–π interactions, have been shown to present covalent characteristics^24^,^25^,^26^,^27^. In the present work, in order to understand how the weak CH-π interaction causes orbital reformation, we systematically examine the electronic properties of the benzene-methane complex, including density of states (DOS), projected density of states (PDOS), and overlap population density of states (OPDOS), at different levels of *ab initio* calculations. The results reveal that strong intermolecular interactions of orbitals occur not only between the occupied π orbital of benzene and the σ orbital of methane but also between the unoccupied orbitals of benzene and methane, resulting in the formation of new molecular orbitals.

## Results

### Orbitals distribution

**Figure 1:**
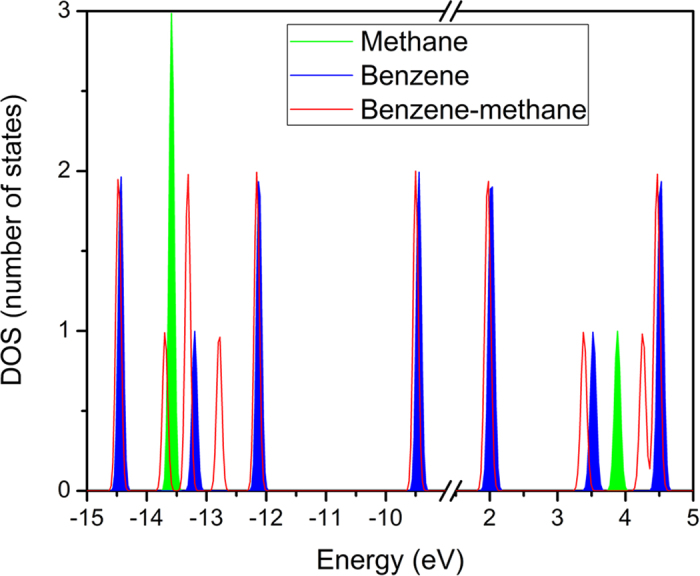
The DOS of benzene, methane, and the benzene-methane complex calculated using ωB97X/6-311G**. The green and blue regions denote the DOS of isolated methane and benzene, respectively. The red line denotes the DOS of the benzene-methane complex.

**Figure 2:**
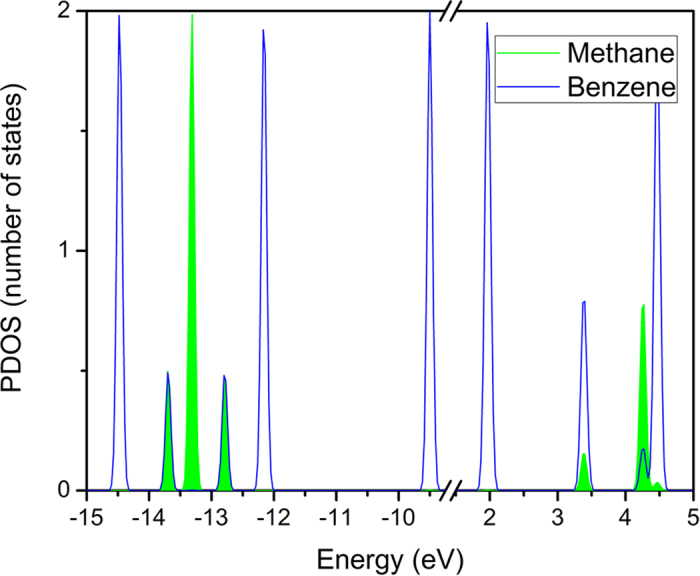
The PDOS of the benzene-methane complex using ωb97x/6-311G**. The green region denotes the PDOS projected on methane and the blue solid line denotes the PDOS projected on benzene.

**Figure 3:**
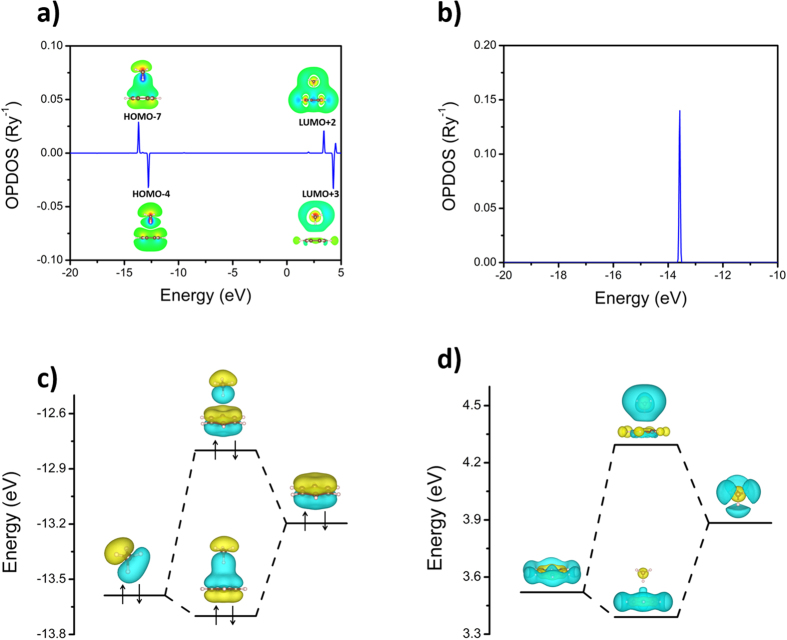
(**a**) OPDOS between benzene and methane for the benzene-methane complex. As a reference, the OPDOS between the carbon and hydrogen atoms in methane is plotted in (**b**). Diagrams (**c**,**d**) respectively show the interaction between two occupied and two unoccupied orbitals of benzene and methane in the benzene-methane complex. The isosurface value is ±0.03 and the blue and yellow colors denote negative and positive value, respectively.

As many experimental and theoretical results have proved that benzene-methane has the on-top type isomer configuration^28^,^29^,^30^, we take this configuration as our model. Previous results demonstrated that the BSSE-corrected intermolecular interaction energy is −1.51 kcal/mol after relaxation and the corresponding distance between the molecular centers is 3.754 Å^23^,^31^. All the occupied and front unoccupied orbitals of isolated benzene, isolated methane and benzene-methane complex were checked (Figs 1S to 3S). In the case of the benzene-methane complex, HOMO-7, HOMO-4, LUMO+2, and LUMO+3 are localized not only at benzene but also at methane. The calculated orbital composition reveals that these orbitals consist of both benzene and methane (Table S1).

### Electronic properties

**Figure 4:**
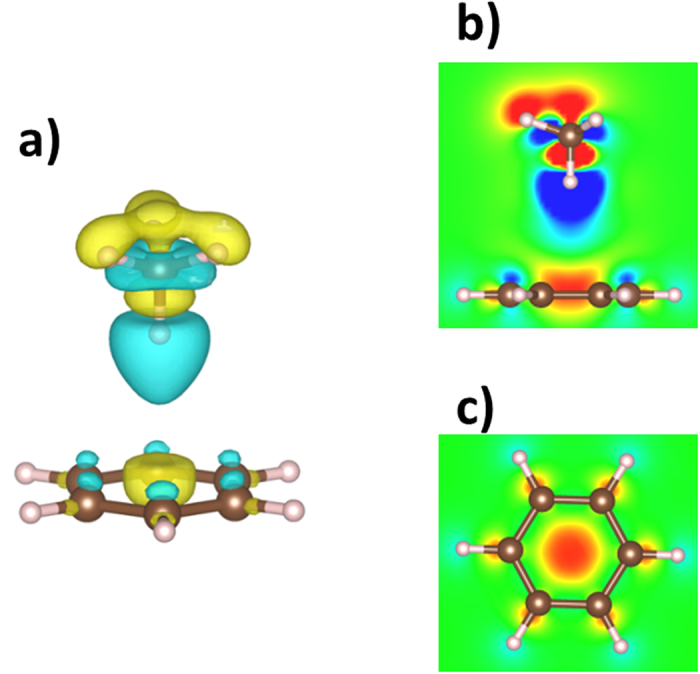
Electron density difference for the benzene-methane complex. (**a**) Plotting in the isosurface. The blue region represents the electron donor, and the yellow region represents the electron acceptor. The isosurface value is ±0.0002. (**b,c**) are the sections along the center of the complex and the plane containing benzene, respectively.

The DOS of the isolated benzene, isolated methane, and benzene-methane complex were calculated using ωB97X/6-311G** (Fig. 1). The results indicate that the energy level of the benzene-methane complex is not a simple superposition between benzene and methane. Under the CH-π interaction, the energy levels of both benzene and methane are changed. Most of the energy levels of the benzene-methane complex are slightly left-shifted (about 0.05 eV) compared with the isolated molecule due to the electrostatic interaction induced by the hydrogen bond interaction between benzene and methane. When the methane molecule gets close to benzene, dispersion interaction results in electric dipole moment, and then the polarized methane molecule causes an electronic field at the benzene molecule, which induces the slight orbital shift in energy of the benzene. Compared with the isolated molecules, the energy levels of the complex located from −14.0 eV to −12.5 eV (HOMO-7 to HOMO-4) and from 3.0 eV to 5.0 eV (LUMO+2 and LUMO+3) are clearly changed. It should be noted that these orbitals are not only from the benzene but also from the methane in this region. By contrast, in the region containing slight changes, the orbitals are localized at benzene or methane and the orbital composition is contributed by benzene or methane. From −14.0 eV to −12.5 eV, there are three degenerate states of isolated methane and one state of isolated benzene. For the benzene-methane complex, there are four states: two degenerate states and two nondegenerate states. The results indicate that orbital interaction occurs between one of the degenerative states of methane and a state of benzene, leading to the change of energy level and a lower degeneracy of the energy level of methane. Regarding the unoccupied energy levels, very similar to the occupied energy levels, there are orbital interactions occurring between the LUMO of methane and a state of benzene (LUMO+2), leading to the change in energy level in the region from 3.0 eV to 5.0 eV. To confirm this result, CCSD(T)/6-311G** calculations for DOS were performed, and these give similar results of ωB97X/6-311G* (Fig. 4S). Around the HOMO and LUMO of methane, intermolecular orbital interaction occurs between benzene and methane, leading to the change of energy level and degeneracy.

To further understand the intermolecular orbital interaction between benzene and methane, we calculate the PDOS of the benzene-methane complex employing ωB97X/6-311G** (Fig. 2). For the ωB97X/6-311G** calculation, the results reveal that both benzene and methane contribute to the LUMO+3, LUMO+2, HOMO-4, and HOMO-7 of the complex , indicating that these orbitals are generated by both of them through intermolecular orbital overlap. The occupied HOMO-4 and HOMO-7 are generated through the intermolecular orbital interaction between the HOMO-2 of benzene and one of the degenerative HOMOs of methane; at the same time, the remaining two degenerative HOMOs of methane form the HOMO-5 and HOMO-6 of the complex. The unoccupied LUMO+3 and LUMO+2 are generated by the LUMO+2 of benzene and the LUMO of methane through intermolecular orbital interaction. One should notice that the orbitals which dominate the intermolecular orbital interaction are energetically close, consistent with the principle of energy closing in the linear combination of atomic orbitals (LCAO) theory. To further confirm the ωB97X/6-311G** results, the PDOS is calculated using different methods and basis sets including the augmented wave (PAW) method^32^,^33^ with London dispersion two-body correction^34^ implemented in the VASP code (Fig. 5S). For CCSD(T)/cc-pVTZ, M062X/6-311G**, MP2/cc-pVTZ and ωB97XD/6-311G** calculations, the results are almost the same except for the values of energy levels and the orbital composition ratio between methane and benzene. Because VASP is not based on local orbital basis sets but on the PAW method, PDOS is calculated by integrating the wave function over spheres with a special radius centered at the atoms’ positions. Therefore, the sum of PDOS is smaller than total DOS for delocalized orbitals. Thus, the value of the DOS of both LUMO+2 and LUMO+3 is very small in PAW calculations due to their strong delocalization. But the overlap of orbitals is similar to the ωB97X/6-311G** results.

## Discussion

OPDOS, also referred to in the literature as crystal orbital overlap population (COOP), is a measure of the overlap strength and an energy resolved quantity which is positive for bonding states and negative for antibonding states^35^,^36^. Using this concept, the OPDOS between benzene and methane was calculated for the benzene-methane complex using the GaussSum program^37^ at the ωB97X/6-311G** level (Fig. 3a). The HOMO-7, HOMO-4, LUMO+2, and LUMO+3 of the complex show strong orbital overlaps between benzene and methane. The values of OPDOS for these four orbitals are 0.029, −0.032, 0.022, and −0.033 Ry^−1^, which represent about 20% of the overlap strength between the carbon and hydrogen atoms in methane (Fig. 3b). To further validate the OPDOS, we calculated its value using different methods and basis sets including CCSD(T)/cc-pVTZ, M062X/6-311G**, MP2/cc-pVTZ, ωB97XD/6-311G**, ωB97X/3-21G and ωB97X/STO-3G, as shown in Fig. 6s. The calculated OPDOS value of HOMO-7 fluctuates from 0.018 Ry^−1^ to 0.028 Ry^−1^ except ωB97X/STO-3G, which clearly shows the existence of orbital overlap. Even using minimal basis set (STO-3G), which is not appropriate to describe intermolecular interaction, the calculated OPDOS value of HOMO-7 is still 0.008 Ry^−1^. The results obviously indicate that the orbital interaction between benzene and methane forms bonding and antibonding orbitals through orbital overlap including both occupied and unoccupied orbitals (Fig. 3c,d), which is very similar to the interaction of interatomic orbitals, although no real chemical bond is formed.

The interaction of intermolecular orbitals not only changes the orbitals’ distribution through orbital overlap but also the energy levels of orbitals in both occupied and unoccupied orbitals (Fig. 3c,d). It is surprising that the total energy rise of 0.568 eV is due to the occupation of the antibonding orbital. However, considering the decrease in the degeneracy of methane’s HOMO, the total energy rise of 1.688 eV is due to the overlap of intermolecular orbitals. The existence of the hydrogen bond between benzene and methane causes the decrease in the other energy levels, finally resulting in −1.51 kcal/mol of intermolecular interaction energy.

The intermolecular interaction between occupied orbitals results in the formation of new molecular orbitals, leading to the rearrangement of the electronic distribution^38^,^39^. The electron density difference for the complex relative to the isolated molecules shows the consequence of orbital interaction between benzene and methane (Fig. 4). Though the amount of the charge transfer is very small (0.002e calculated at the MP2/6-311G** level) from benzene to methane^29^, an obvious transfer can be found within the molecule. Within the methane molecule, the charge transfer results in the polarization of methane toward benzene. Interestingly, there is an electronic accumulation in the center of benzene which results from the intermolecular orbital interaction.

## Conclusion

We report for the first time the interaction of intermolecular orbitals between benzene and methane. Using *ab initio* calculations based on different methods and basis sets, we calculate the DOS, PDOS, and OPDOS and provide diagrams of the intermolecular interaction of orbitals. Our results demonstrate an obvious intermolecular orbital interaction between benzene and methane. The intermolecular interaction of orbitals forms bonding and antibonding orbitals through orbital overlap and increases the total energy of the complex because of the occupation of the antibonding orbital. At the same time, the intermolecular orbital interaction results in the rearrangement of electronic distribution. Our finding extends the concept of orbital interaction and provides a deeper understanding of the CH-π hydrogen interaction between benzene and methane. Due to the wide existence of CH-π interaction in biological and organic molecules, the concept of intermolecular orbital interaction should help the understanding and design of new molecules with weak interaction.

## Methods

### Computational methods

The *ab initio* calculations about structural relaxations and electronic properties are carried out with the Gaussian 09 suite of programs (G09d01 version)^40^. A long-range corrected hybrid density functional ωB97X 41 has been used to explore the configuration and interaction energy at the ground state. The ωB97X hybrid density functional includes 100% long-range exact exchange, applying generalized gradient expressions for short-range exchange. The basis set superposition error (BSSE)^42^ was corrected for all calculations with the counterpoise method^43^,^44^. For comparison, a single point CCSD(T)^45^ calculation has been used to show the impact on DOS of intermolecular interaction in different calculation methods. To confirm the interaction of intermolecular orbitals between benzene and methane, a PDOS calculation is performed in the framework of the density functional theory within the generalized gradient approximation Perdew-Burke-Ernzerhof (GGA-PBE)^46^, as implemented in the VASP code^47^. The projector augmented-wave (PAW)^32^,^33^ pseudo-potentials are used to describe the ionic potentials. The cutoff energy (800 eV) is used for the expansion of the wavefunction into plane waves at Γ points. In order to account for long-range corrections of intermolecular interactions, we adopted the London dispersion two-body correction to the DFT approximation (DFT-D3)^34^.

## Additional Information

**How to cite this article**: Li, J. and Zhang, R.-Q. Strong orbital interaction in a weak CH-π hydrogen bonding system. *Sci. Rep.* **6**, 22304; doi: 10.1038/srep22304 (2016).

## Supplementary Material

Supplementary Information
