# Supplementary material for: Strong orbital interaction in a weak CH-π hydrogen bonding system
Source: Sci Rep. 2016 Mar 1;6:22304. doi: 10.1038/srep22304 (PMC4772115; doi:10.1038/srep22304)
Supplement: Supplementary Information [file srep22304-s1.pdf]

Supplementary information for

Strong orbital interaction in a weak CH-π hydrogen bonding system

Jianfu Li, Rui-Qin Zhang*

Department of Physics and Materials Science, City University of Hong Kong, Hong Kong SAR, China

Labels of orbitals	LUMO	HOMO	HOMO-1	HOMO-2	HOMO-3	HOMO-4
Orbitals	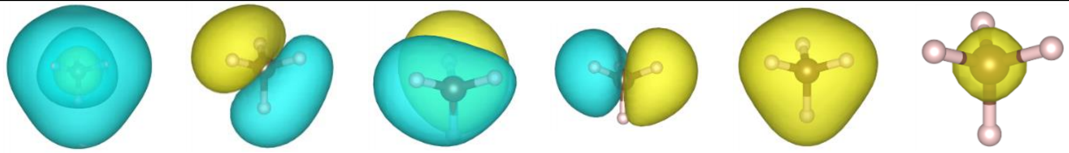					

Figure 1S. Molecular orbitals of the isolated methane calculated by ωB97X/6-311G** basis sets. The isosurface value is ±0.03 and the blue and yellow colors denote negative and positive value, respectively. Small and large balls denote hydrogen and carbon atoms, respectively.

Labels of orbitals	LUMO+2	LUMO+1	LUMO	HOMO	HOMO-1	HOMO-2
Orbitals	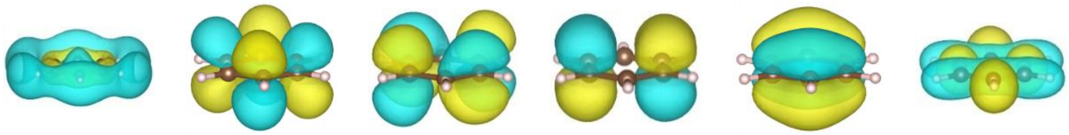					
Labels of orbitals	HOMO-3	HOMO-4	HOMO-5	HOMO-6	HOMO-7	HOMO-8
Orbitals	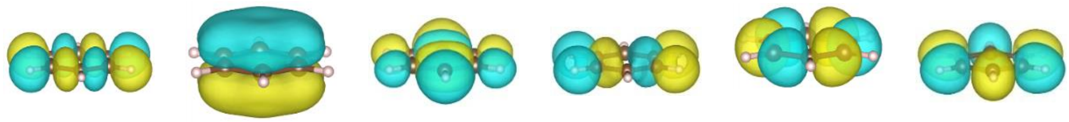					
Labels of orbitals	HOMO-9	HOMO-10	HOMO-11	HOMO-12	HOMO-13	HOMO-14
Orbitals	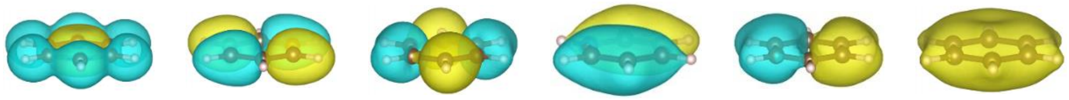					
Labels of orbitals	HOMO-15	HOMO-16	HOMO-17	HOMO-18	HOMO-19	HOMO-20
Orbitals	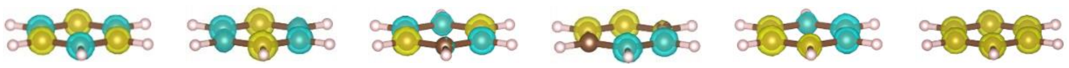					

Figure 2S. Molecular orbitals of the isolated benzene calculated by using ωB97X/6-311G** basis sets. The isosurface value is ±0.03 and the blue and yellow colors denote negative and positive value, respectively. Small and large balls denote hydrogen and carbon atoms, respectively.

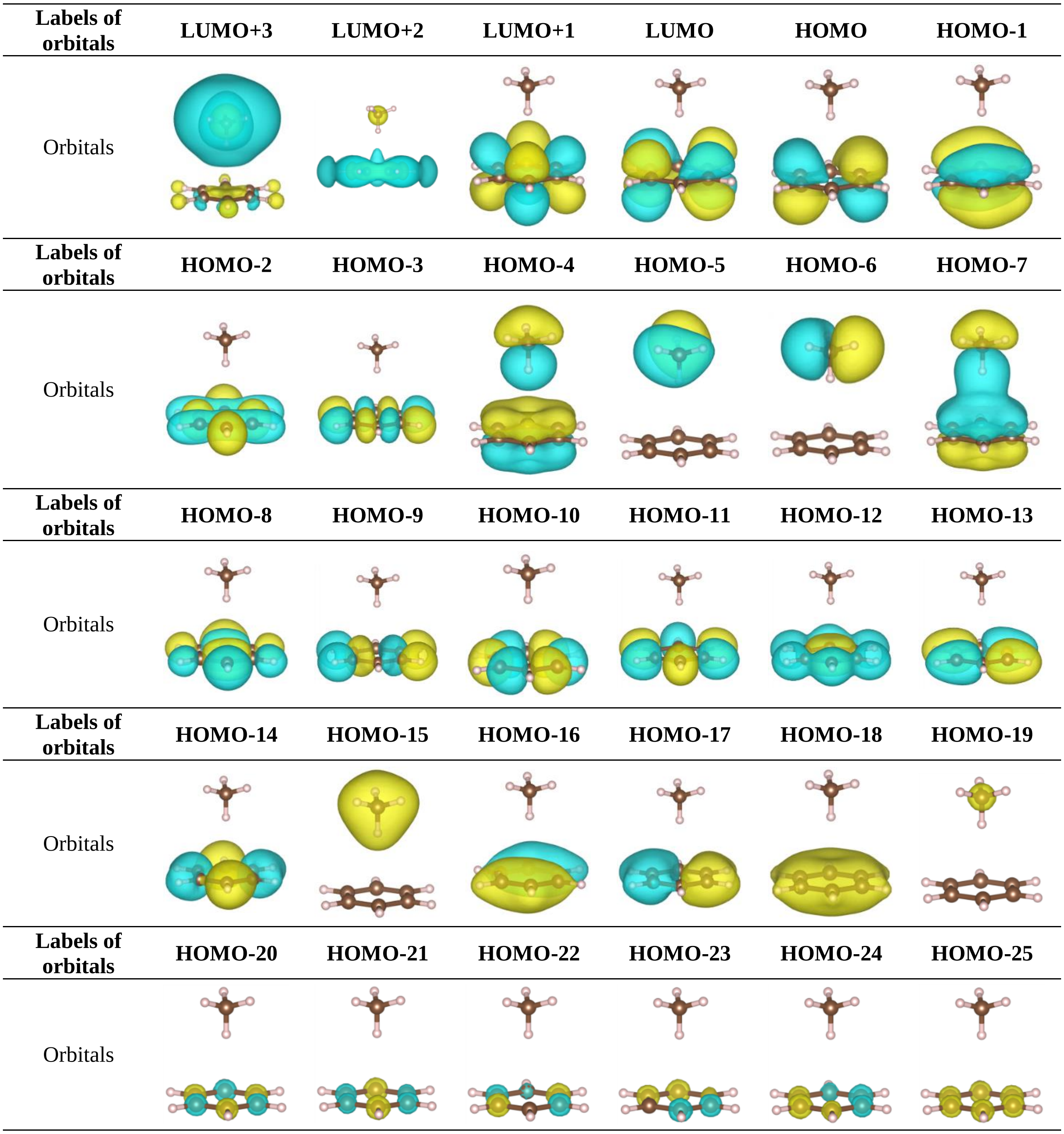

Figure 3S. Molecular orbitals of the benzene-methane complex calculated by ωB97X/6-311G** basis sets. The isosurface value is ±0.03 and the blue and yellow colors denote negative and positive value, respectively. Small and large balls denote hydrogen and carbon atoms, respectively.

Table 1S. Orbital composition of benzene-methane complex.

	HOMO-7	HOMO-6	HOMO-5	HOMO-4	HOMO-3	HOMO-2	HOMO-1	HOMO	LUMO	LUMO+1	LUMO+2	LUMO+3
CH_4	48%	100%	100%	52%	0%	0%	0%	0 %	0%	0%	19%	81%
C_6H_6	52%	0%	0%	48%	100%	100%	100%	100%	100%	100%	81%	19%

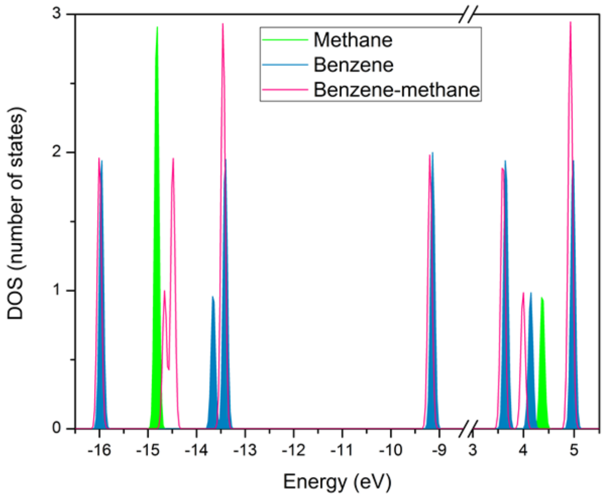

Figure 4S. The DOS of benzene, methane and benzene-methane complex calculated by CCSD(T)/6-311G** basis sets. Green and blue regions denote the DOS of isolated methane and benzene, respectively. Red solid line denotes the DOS of benzene-methane complex.

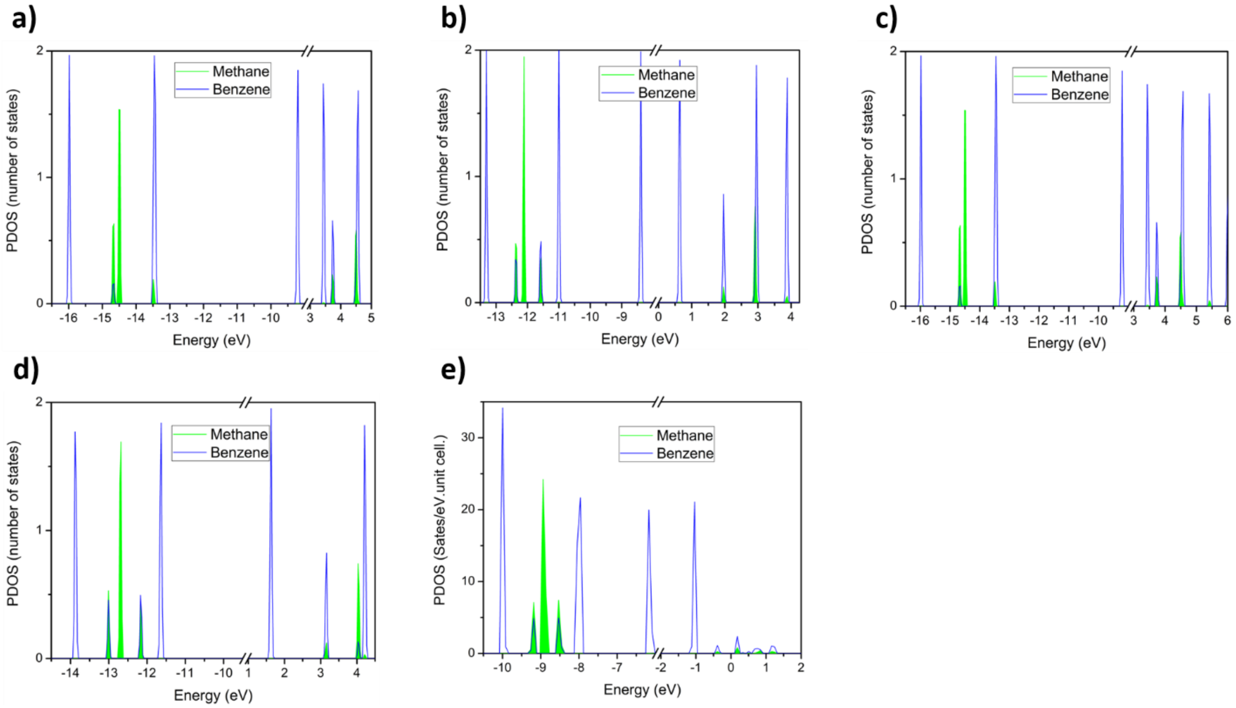

Figure 5S. The PDOS of benzene-methane complex using different methods and basis sets. a) CCSD(T)/cc-pVTZ, b) M062X/6-311G**, c) MP2/cc-pVTZ, d) ωB97XD/6-311G**, e) PAW method with London dispersion two-body correction. Green region denotes the PDOS projected on methane and blue solid line denotes the PDOS projected on benzene.

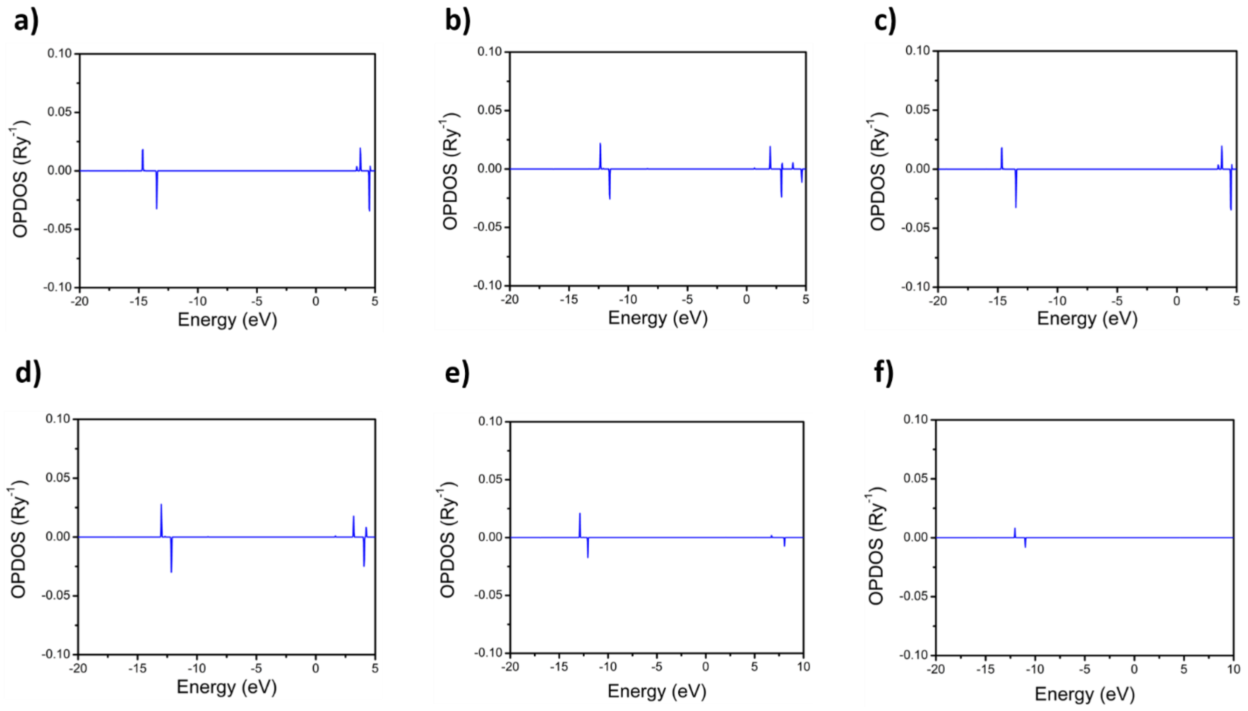

Figure 6S. The OPDOS between benzene and methane for the benzene-methane complex using different methods and basis sets. a) CCSD(T)/cc-pVTZ, b) M062X/6-311G**, c) MP2/cc-pVTZ, d) ωB97XD/6-311G**, e) ωB97X/3-21G and f) ωB97X/STO-3G.
